# Is a Higher Amniotic Fluid Viral Load Associated with a Greater Risk of Fetal Injury in Congenital Cytomegalovirus Infection—A Systematic Review and Meta-Analysis

**DOI:** 10.3390/jcm13072136

**Published:** 2024-04-07

**Authors:** Noa Gilad, Swati Agrawal, Eleni Philippopoulos, Kellie E. Murphy, Shiri Shinar

**Affiliations:** 1 Department of Obstetrics and Gynecology, University of Toronto, Toronto, ON M5G 1E2, Canada; noa.gilad@sinaihealth.ca (N.G.); eleni.philippopoulos@mcgill.ca (E.P.); kellie.murphy@sinaihealth.ca (K.E.M.); 2 Department of Obstetrics and Gynecology, University of McMaster, Hamilton, ON L8S 4K1, Canada; agraws5@mcmaster.ca

**Keywords:** CMV, viral load, amniotic fluid, symptoms prediction, quantification, ultrasound

## Abstract

**Background**: Numerous studies have aimed to predict prenatal and neonatal outcomes for pregnancies complicated by congenital cytomegalovirus (CMV). Presently, assessing CMV severity prenatally relies largely on fetal imaging. A controversy exists regarding CMV viral load (VL) and its association with fetal and neonatal sequelae. **Objective**: To perform a systematic review and meta-analysis investigating the association between CMV DNA VL in amniotic fluid and fetal and neonatal outcomes in pregnancies with congenital CMV. **Results**: All cohort, case-control and observational studies that compared outcomes of fetuses with congenital CMV and provided information on individual patient CMV VL quantified in copies per milliliter (c/mL) from inception to January 2023 were included, with no geographical or language restrictions. A total of 1251 citations were reviewed with eight studies meeting inclusion criteria and included in meta-analysis. Affected pregnancies had a higher VL in the amniotic fluid compared to those unaffected with a mean difference of 2.2e+7 (range 1.5e+7 to 2.8e+7). In subgroup analysis, the VL was significantly higher in the fetuses, with imaging findings related to CMV compared to asymptomatic fetuses with a mean difference of 4.1e+7 (95% CI 2.8e+7–5.4e+7). However, among babies with congenital CMV, the VL was not significantly different between symptomatic and asymptomatic babies. **Conclusions**: Amniotic fluid CMV VL is associated with fetal sequalae in congenital CMV, with a higher VL conferring a greater risk for prenatal injury.

## 1. Introduction

Each year, in the United States, congenital cytomegalovirus (CMV) occurs in about 1 out of 200 infants [1], making it the most prevalent prenatal infection and a major contributor to infectious-related disabilities. The most severe outcomes of congenital infection include permanent hearing and vision impairments, microcephaly, and associated neurological disabilities [2]. Research has identified an inverse relationship between infection rates and the impact on fetuses across trimesters [3]. Numerous studies have aimed to predict prenatal and neonatal outcomes for infected fetuses. Presently, assessing CMV severity during pregnancy relies largely on fetal imaging, utilizing targeted ultrasound, neurosonography, and Magnetic resonance imaging (MRI). However, even these modalities have proven insufficient, as demonstrated in a 2021 meta-analysis [4]. Notably, even when ultrasound or MRI results appear normal, 1.5% of infants still exhibit symptomatic infection, 3.1% display neurodevelopmental anomalies, and 6.5% experience hearing issues.

Given this limitation of prenatal imaging in predicting outcome, and the possibility that injury may at times be detected only late in pregnancy, the search for quantifiable potential outcome predictors is invaluable. Although CMV glycoprotein B genotype was initially thought to influence infection severity [5,6], subsequent attempts to confirm this have been largely unsuccessful [7]. Additional research has suggested that fetal platelet levels [7,8], CMV DNA concentrations in fetal blood [7,8,9], a specific genotype of the NFKB1 polymorphism [10], and amniotic fluid peptides [11,12] are all associated with a symptomatic status at birth. If proven reliable in future robust research, these techniques will still require invasive fetal blood sampling (for platelet levels and CMV DNA concentrations) and specific laboratory platforms for proteome analysis. A simpler, largely available tool for predicting outcome prenatally relies on amniotic fluid CMV DNA quantification. Indeed, a high CMV viral load (VL) has been associated with a higher risk of clinical sequelae in some studies [13,14,15,16], but not others [7,17,18]. In light of this controversy, we aimed to perform a systematic review and meta-analysis investigating the association between CMV DNA VL in amniotic fluid and fetal, as well as neonatal, outcome in pregnancies complicated by congenital CMV.

## 2. Materials and Methods

The study protocol for this systematic review was developed and registered with PROSPERO (registration number: CRD42023389109) prior to undertaking the search, selecting the studies, and extracting the data. This systematic review was reported in accordance with the PRISMA guidelines.

No financial or nonfinancial support or sources were used during the review.

## 3. Inclusion and Exclusion Criteria

All cohort, case-control and observational studies that compared outcomes of fetuses with congenital CMV that provided information on individual patient amniotic fluid CMV VL, quantified in copies per milliliter (c/mL), were included. We excluded conference abstracts, case reports with fewer than five subjects, editorials, letters, and personal communications. Studies with no comparison of VL or fetal and/or neonatal outcome or in which the VL was reported in GE/mL or IU/mL were excluded. Research in which antiviral therapy was initiated but was administered unevenly among different groups were excluded as well due to potential confounding.

## 4. Search Strategy

A comprehensive search strategy was developed by an information specialist (EP). The following databases were searched from inception to 6 December 2022: Medline (Ovid), PubMed (non-Medline records only), Embase (Ovid), Web of Science (Clarivate), CINAHL (Ebsco), Cochrane Database of Systematic Reviews (Ovid), Cochrane Central Database of Controlled Trials (Ovid), and Global Index Medicus. The first 200 results from Google Scholar were exported to identify any grey literature. A search was also conducted in ClinicalTrials.gov to identify any ongoing trials. The searches in the Cochrane Database of Systematic Reviews and the Cochrane Central Database of Controlled Trials were run simultaneously. Reference lists were of relevant articles that were consulted and relevant articles that were identified. Searches for all databases can be found in Supplementary Materials. No limits relating to language or date were used when searching. Records were sorted and screened using Endnote and Covidence, respectively, and deduplicated automatically using Covidence.

Abstracts underwent screening, and relevant publications underwent thorough review by two autonomous investigators (NG and SS), utilizing Covidence software (Covidence systematic review software 2023, Veritas Health Innovation, Melbourne, Australia. Available at www.covidence.org) for systematic review management. A third investigator (SA) acted as an adjudicator in instances where consensus was lacking. In cases where the same cohort appeared in multiple studies, only the larger cohort was incorporated into the analysis. The characteristics of studies included in the meta-analysis can be seen in Table 1.

## 5. Quality Analysis

Two autonomous investigators (NG and SS) conducted quality analysis utilizing the study quality assessment tool for case-control studies offered by the National Heart, Lung, and Blood Institute of the National Institutes of Health (NIH) [24]. The analysis addressed twelve distinct research inquiries concerning the study’s objectives, the composition and sizes of the study and control populations, the accuracy of population identification, random selection procedures, timing of exposure, blinding techniques, and confounding variables. Studies were rated as good, fair, or poor taking into account these measured variables as well as overall subjective reviewer assessment, as defined for this tool (Table 2).

## 6. Statistical Analysis

All relevant data from individual studies for specified outcomes were extracted and entered into a spreadsheet based on the Cochrane data extraction tool. Data were extracted from all studies for affected and unaffected fetuses with congenital CMV. Amniotic fluid VL mean and standard deviations were collected if given in the study or if not, they were calculated from the individual patient data provided in the study. A random effects Der Simoniam Laird model was used to calculate the weights of the individual studies and to generate a summary estimate of mean difference and 95% confidence intervals (CI) between the affected and unaffected fetuses for the VL in the amniotic fluid. This was graphically depicted using a forest plot. The heterogeneity was calculated using the I2 statistic to delineate the variability between the studies, which could be attributed to methodology. Funnel plots were utilized to evaluate publication bias and were tested for asymmetry using Egger’s test. Subgroup analysis for fetal and neonatal findings was performed to study the association between VL and outcomes in CMV. Studies that provided both fetal and neonatal outcomes as independent cohorts [12] were depicted as two separate studies for the purpose of the analysis. The Egger’s meta-regression model helped to assess the magnitude and statistical significance of the relationship between observed effect sizes and the size of studies. All analyses were performed using StataSE version 16 (StataCorp, College Station, TX, USA).

## 7. Results

**Figure 1:**
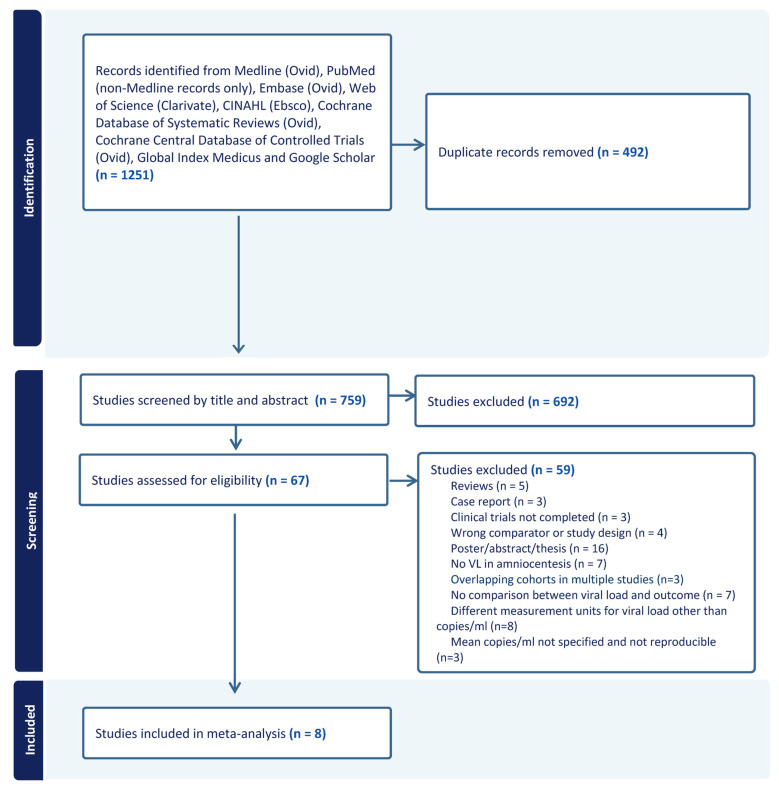
Flow chart of studies included in meta-analysis.

A total of 1251 citations were identified from our search, 492 of which were duplicates. The remaining 759 were screened for title and abstract, of which 692 did not meet inclusion criteria and were therefore excluded. Sixty-seven studies underwent full text review for assessment of eligibility and 59 of these were excluded for various reasons, as listed in Figure 1. Eight studies fulfilled inclusion criteria and were included in our meta-analysis [11,12,17,19,20,21,22,23]. These studies were published between 2009 and 2023 and originated from Europe (six from Italy, one from France, and one from Belgium) and Asia (one from Israel). Pooled together these studies included a total of 443 fetuses with congenital CMV in pregnancy. Five studies reported on fetal outcomes [11,12,19,20,21], with a total of 272 fetuses. Three studies reported on postnatal outcomes [12,17,23], with a total of 126 neonates. Only one study reported on both fetal and neonatal outcomes with 45 individuals [12]. One study [22] did not differentiate between fetal and neonatal outcomes.

All included studies were retrospective cohort studies. The characteristics of the studies are presented Table 1. Notably, data regarding gestational age at amniocentesis, mean VL, fetal outcome as determined by prenatal imaging (US and/or MR), neonatal outcome as well as length of neonatal follow up, were retrieved.

The quality analysis revealed three studies of good quality and five studies of fair quality. None of the included studies were deemed to be of poor quality (Table 2).

## 8. Outcome Definitions

Both fetal and neonatal outcomes were variable. No uniform definition was used for affected fetuses across the studies beyond findings on ultrasound and/or MRI. Similarly, no uniform definition was used to describe symptomatic neonates or differentiate between symptom severity. One study differentiated between fetal CNS and non CNS findings [11], while the others did not. CNS findings included microcephaly, periventricular pseudocysts, ventriculomegaly with intracranial calcifications, malformations of cortical development, destructive encephalopathy, intracranial calcifications in the basal ganglia or germinal matrix, and multiple CNS anomalies, not otherwise specified. Extra-CNS findings included hepatomegaly, ascites, echogenic bowel, liver calcifications, fetal growth restriction, pericardial effusion, placentomegaly, and fetal demise. The prenatal diagnosis was made using ultrasound alone [17,20] or a combination of ultrasound and MRI [11,12,19,21,23].

Neonatal findings, as described in the studies, included preterm birth, small for gestational age, petechiae or purpura, hepatosplenomegaly, CNS abnormalities, elevated liver enzymes, thrombocytopenia or conjugated hyperbilirubinemia, neurologic disturbances, delays in psychomotor and/or mental developmental status, and CMV-related audiological or visual problems. The follow-up period ranged from six months [19] to six years [23].

## 9. Risk of Adverse Perinatal Outcome

**Figure 2:**
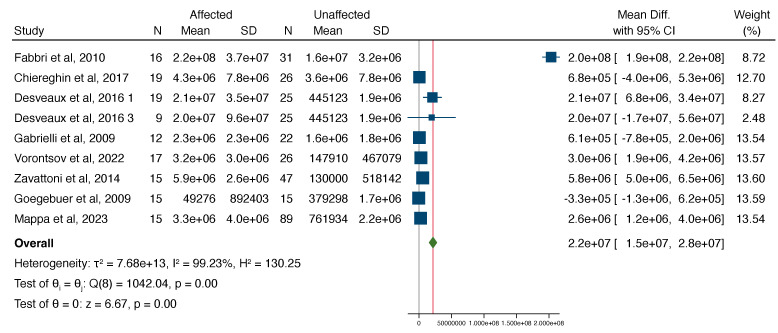
Forest plot showcasing the standardized mean difference in the mean viral load between cases affected and not affected by CMV [11,12,17,19,20,21,22,23].

**Figure 3:**
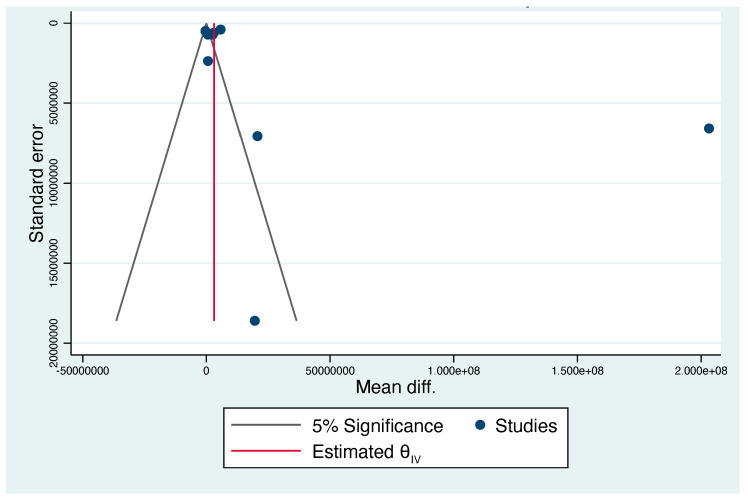
Funnel plot of difference in the mean viral load between cases affected and not affected by CMV. Publication bias was observed.

Of all pregnancies with congenital CMV, those affected had a significantly higher VL in the amniotic fluid compared to those unaffected, with a mean difference of 2.2e+7 (range 1.5e+7 to 2.8e+7, Figure 2). The funnel plot showed evidence of publication bias (Figure 3). Egger’s test suggested that smaller studies did not tend to show different results if compared with larger studies, as the CI of the intercept included the value zero with a *p*-value of 0.099.

The study by Fabri et al. was detected as an outlier and therefore a “leave-one-out” analysis was performed. The results of the meta-analysis did not change significantly, with an overall mean difference in the viral load being 2.7e+6 (95% CI 3.3e+5 to 5.1e+6), with a significantly higher load in affected cases than unaffected cases.

**Figure 4:**
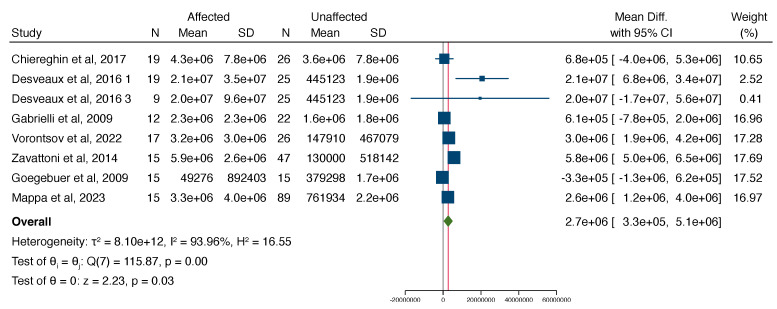
Forest plot showcasing the standardized mean difference in the mean viral load between cases affected and not affected by CMV excluding the study by Fabri et al. as it was an outlier [11,12,17,20,21,22,23].

**Figure 5:**
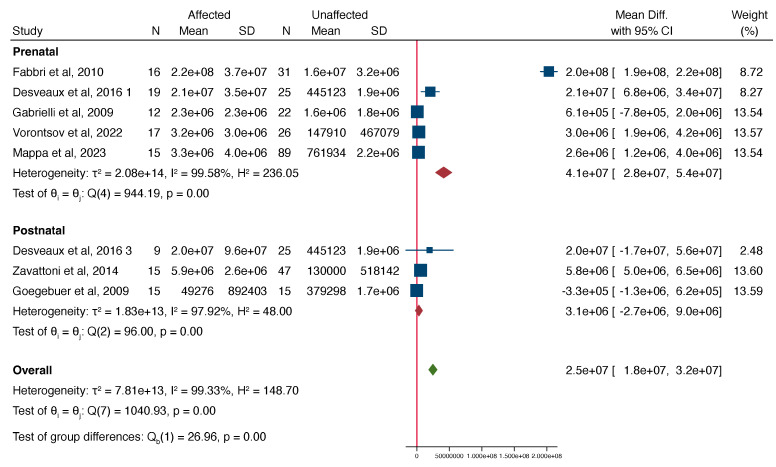
Forest plot showcasing the standardized mean difference in the mean viral load in two subgroups: prenatal and postnatal cases; between cases affected and not affected by CMV [11,12,17,19,20,21,23].

In a subgroup analysis, assessing the association between amniotic fluid VL and fetal and neonatal findings, the VL was significantly higher in the fetuses, with imaging findings related to CMV compared to asymptomatic fetuses with a mean difference of 4.1e+7 (95% CI 2.8e+7–5.4e+7), but the VL was not significantly different between symptomatic neonates compared to asymptomatic neonates with congenital CMV (Figure 4 and Figure 5).

## 10. Discussion

### 10.1. Main Findings

In this systematic review and meta-analysis, we found that the CMV VL in the amniotic fluid, detected using quantitative PCR, is associated with fetal sequalae in congenital CMV, with a higher VL conferring greater risk for prenatal injury. In neonates with congenital CMV, however, we did not find an association between the amniotic fluid VL and neonatal sequelae.

### 10.2. Strengths and Limitations

Our meta-analysis stands out for its thorough literature search, which encompassed nine search engines, including the gray literature, effectively mitigating potential publication bias. We included all study designs without language or geographic restrictions. Notably, our search terms did not define outcomes, enhancing the robustness of our analyses. To address significant heterogeneity among studies, we employed a random-effects Der Simonian-Laird model [25].

Nonetheless, important limitations should be recognized. All studies included were observational, with no randomized controlled trials on this topic. Observational studies are prone to confounding and bias because the comparison groups may be different in characteristics that are associated with the outcomes studied. Indeed, none of the studies controlled for potential confounders, such as gestational age at the time of supposed cytomegalovirus seroconversion as well as timing of amniocentesis. It is well-established that the likelihood of fetal sequelae correlates with the time of CMV seroconversion and fetal infection, with first trimester infection associated with the highest risk of fetal stigmata [26,27]. As such, it is plausible that the effect of the amniotic VL is also dependent on the gestational age at infection. In most studies included in this meta-analysis, the gestational age at sampling ranged between 19 and 22 weeks; however, in some studies an amniocentesis was performed as late as 27 [11], 29 [12], and even 32 weeks [17]. Furthermore, all studies were retrospective cohort studies and, therefore, fetal anomalies were already present at the time of amniocentesis and VL quantification, making causation difficult to establish. Cases where a VL is prudent, i.e., in serologies indicative of recent CMV seroconversion with normal fetal anatomy, were only partly represented in the studies [11,20,21], as many of the amniocenteses were performed due to existing ultrasound findings. The definition of the outcomes assessed was nonuniform across all studies and not all studies provided an outcome definition [22]. While most studies described prenatal ultrasound or MRI findings, neonatal outcomes were not always described [20,21,22]. Additionally, only three studies reported and defined neonatal outcomes, but differed in the neonatal follow-up time periods, limiting the reliability of reporting neonatal and childhood outcomes. Some studies did not specify length of follow up [20,21], some were followed for up to six months after birth [19], while others were for up to six years [23]. This is an important limitation since hearing loss may be progressive and detected only in later childhood years [28]. It is well-established that the primary determinant of adverse perinatal outcome is cerebral involvement [29]. Therefore, it would have been prudent to stratify our results and report the association between VL and cerebral injury. Unfortunately, all studies except one [11] assessed prenatal findings as a whole and did not differentiate between CNS and non-CNS lesions. Lastly, as with all meta-analyses, the results are dependent on the methodologic quality of the studies included. Reassuringly, here, the quality was fair to good and none of the studies included were deemed poor quality.

### 10.3. Implications of Findings

Primary CMV in the first trimester is associated with adverse perinatal outcome in 1 of 10 affected fetuses [30]. Of those with symptomatic infection at birth, 50% will have irreversible sequelae, primary sensorineural hearing loss (SNHL), or neurodevelopmental impairment [31]. One of the main challenges in prenatal counseling of expectant parents is determining who among infected fetuses will be subject to poor outcome. Currently, antenatal prognosis is mainly dependent on the presence of imaging findings on neurosonography or fetal MRI. However, these findings are nonspecific, may appear late in gestational age, and their absence does not guarantee a favorable outcome, as developmental impairment and auditory issues can still occur [29]. The understanding that findings can be detected at any time in pregnancy, including late in the third trimester [30,31], confers great anxiety and emotional turmoil for expecting parents. Thus, attempts have been made to detect laboratory predictors of neonatal injury in fetal blood or amniotic fluid. Fetal thrombocytopenia and fetal serum levels of beta-2 microglobulin acquired through fetal blood sampling have been suggested to precede the development of brain lesions [8,19,32]. In the amniotic fluid, CMV peptidome analysis yielded 34 peptides associated with a higher risk of symptomatic neonates [12]. These studies serve as evidence for the need to find quantifiable objective antenatal measures to predict neonatal outcome. Our finding that a correlation exists between the amniotic fluid VL and the likelihood of developing prenatal injury is promising as it may serve as a tool to help stratify risk in cases of established congenital CMV. If future prospective large-scale studies find a similar association, we may be able to reassure parents when presented with a low VL. Moreover, although prenatal treatment of fetal CMV is controversial [33,34,35], the use of VL as a tool to stratify candidates who may benefit from antenatal treatment or as a marker of response to treatment is an additional direction for future research.

## 11. Conclusions

While a higher amniotic fluid VL is associated with a greater risk of fetal injury, there is insufficient evidence to determine its association with neonatal disease. Our findings may, in part, be due to bias including uncontrolled or residual confounding. Additionally, the lack of association between VL and neonatal outcomes may be due to higher incidence of terminations in pregnancies with a higher VL and more severe imaging findings or later onset of SNHL beyond the period of follow up. Care providers to patients with fetal CMV should be mindful of this association; however, it would be premature, on the basis of these preliminary results, to attempt to prognosticate fetal sequalae or determine need for future surveillance. Further study is indicated to assess pregnancy outcomes in pregnancies complicated by congenital CMV with more robust control for potential confounding variables before clinical recommendations can be made.

## Figures and Tables

**Table 1:** Characteristics of studies included in the meta-analysis.

Characteristics of Studies Included in the Meta-Analysis													
Study and First Author	Country of Origin	Years	Study Design	Total (n)	Sym (n)	Mean VL in Sym c/mL	Asym (n)	Mean VL in Asym c/mL	Timing of Amniocentesis (wks)	CNS Findings	Extra CNS Findings	Diagnostic Tool	Neonatal Outcome and Length of Follow Up
**Studies reporting fetal outcomes only**													
Fabri 2010 [19]	Italy	1995–2009	RS	47	16	2.2e+08	31	1.6e+07	NOS	VM, microcephaly, MRI alteration	echogenic bowel, Ascites, hyperechogenic liver, pelvic effusion, enlarged placenta	US and MRI	N/A
Gabrielli 2009 [20]	Italy	NOS	RS	34	12	2.3e+06	22	1.6e+06	20–21	VM, periventricular heterogenicity, hydrocephalus	FGR, echogenic bowel, hepatomegaly	US	N/A
Vorontsov 2022 [11]	Israel	NOS	RS	43	17	3.2e+06	26	147,910	20–23 (and at least 6 wks after the assumed time of infection	US—temporal cysts, hyperechogenic ventricular wall, cortical and thalamic calcifications, VM > 15 mm, ACC, polymicrogyria, head circumference < 2 SD, white matter cystic lesions, periventricular hyper echogenicity and cysts, small cerebellum, intracerebral calcifications, porencephalic cyst in the occipital lobe; periventricular calcifications, intraventricular adhesions, temporal cysts, mild VM, hypoplasia of corpus callosum, enlarged cisterna magna. MRI—temporal cysts, polymicrogyria, occipital and temporal cysts	hydrops (ascites, pericardial effusion, cardiomegaly) echogenic bowel, liver calcifications, placentomegaly,	US and MRI	N/A
Mappa 2023 [21]	Italy	2012–2021	RS	104	15	3.3e+06	89	761,934	Symptomatic—20.6 ± 1.18 Asymptomatic 20.5 ± 1.39 (*p* = 0.81)	microcephaly, periventricular pseudo cyst, VM with intracranial calcifications, malformations of cortical development, destructive encephalopathy, intracranial calcifications in basal ganglia or germinal matrix	NOS	US and MRI	N/A
**Studies reporting fetal and neonatal outcomes**													
Chiereghin 2017 [22]	Italy	NOS	RS	45	19	4.3e+06	26	3.6e+06	20–21 (and at least 6–8 wks after maternal infection)	NOS	NOS	NOS	NOS
Desveaux 2016 ^1^ [12]	France	NOS	RS Fetal comparison	44	19	2.1e+07	25	445,123	Symptomatic—mean 23 (17–29) Asymptomatic—mean 22 (range 17–29) (*p* = 0.4)	lissencephaly, diffuse white matter lesions, intraventricular adhesions, microcephaly, intra-cerebral clastic lesions, multiple intracerebral calcifications, hypoplasia of the corpus callosum, VM, PVL, lateral ventricle partitioning, periventricular necrosis.	hepatomegaly, ascites, echogenic bowel, liver calcifications, FGR, pericardial effusion, placentomegaly	US and MRI	N/A
Desveaux 2016 ^2^ [12]			RS Neonatal comparison	34	9	2.0e+07	25	445,123		N/A	N/A	US and MRI	Severe psychomotor delay, epilepsy, bilateral hearing loss. 36 m follow-up
**Studies reporting neonatal outcomes only**													
Zavattoni 2014 [23]	Italy	1995–2009	RS	62	15	5.9e+06	47	130,000	Median 21 (range 18–35) with a time interval of 13 weeks (range 2–14) between maternal infection and amniocentesis	N/A		US and MRI	chorioretinitis, hearing loss, petechiae, hepatomegaly, hepatitis, thrombocytopenia, prematurity, anemia, SGA, hypotonia. 6y follow-up
Goegebuer 2009 [17]	Belgium	2002–2006	RS	30	15	49,276	15	379,298	15.5−32.1	N/A	N/A	US	preterm birth, SGA, petechiae or purpura, hepatosplenomegaly, CNS abnormalities, elevated liver enzymes, thrombocytopenia or conjugated hyperbilirubinemia, neurologic disturbances, delays in psychomotor and/or mental developmental status, and CMV-related audiological or visual problem. Up to 3 yrs of follow-up
Total (n)				443	137		306						

Fetal and neonatal findings are described only if quantitative analysis could be performed. Desveaux 2016 ^1^ pertains to a cohort of fetuses with CMV; Desveaux 2016 ^2^ pertains to a cohort of neonates with congenital CMV; Sym—symptomatic; Asym asymptomatic; VL—viral load; CNS—central nervus system; RS—retrospective; e—10^6^; NOS—not otherwise specified; N/A—not applicable; US—ultrasound; MRI—magnetic resonance imagining; VM –ventriculomegaly; wks—weeks of gestation; IUFD—intrauterine fetal demise; TOP—termination of pregnancy; m—months; PVL—periventricular leukomalacia; CMV—cytomegalovirus, ACC—agenesis of corpus callosum, FGR—fetal growth restriction; SGA—small for gestational age.

**Table 2:** Studies included quality assessment.

Study	Population Selection								Exposure				Overall
	**Was the research question or objective in this paper clearly stated and appropriate?**	**Was the study population clearly specified and defined?**	**Did the authors include a sample size justification?**	**Were controls selected or recruited from the same or similar population that gave rise to the cases (including the same timeframe)?**	**Were the definitions, inclusion and exclusion criteria, algorithms or processes used to identify or select cases and controls valid, reliable, and implemented consistently across all study participants?**	**Were the cases clearly defined and differentiated from controls?**	**If less than 100 percent of eligible cases and/or controls were selected for the study, were the cases and/or controls randomly selected from those eligible?**	**Was there use of concurrent controls?**	**Were the investigators able to confirm that the exposure/risk occurred prior to the development of the condition or event that defined a participant as a case?**	**Were the measures of exposure/risk clearly defined, valid, reliable, and implemented consistently (including the same time period) across all study participants?**	**Were the assessors of exposure/risk blinded to the case or control status of participants?**	**Were key potential confounding variables measured and adjusted statistically in the analyses? If matching was used, did the investigators account for matching during study analysis?**	
Fabri [19]	3	3	2	3	3	3	1	2	3	3	2	2	Fair
Chiereghin [22]	3	3	2	3	3	2	1	2	3	3	2	2	Good
Desveaux [12]	3	3	2	3	3	3	1	2	3	3	2	2	Good
Gabrielli [20]	3	3	2	3	3	3	1	2	3	3	2	2	Fair
Vorontsov [11]	3	3	2	3	3	3	1	2	3	3	2	2	Good
Zavatoni [23]	3	3	2	3	3	3	1	2	3	3	2	2	Fair
Goegebuer [17]	3	3	2	3	3	3	1	2	3	3	2	2	Fair
Mappa [21]	3	3	2	3	3	3	1	2	3	3	2	2	Good

(3 Green—yes; 2 Orange—no; 1 Red—not applicable).

## Supplementary Materials

The following supporting information can be downloaded at: https://www.mdpi.com/article/10.3390/jcm13072136/s1.
