# Supplementary material for: Is a Higher Amniotic Fluid Viral Load Associated with a Greater Risk of Fetal Injury in Congenital Cytomegalovirus Infection—A Systematic Review and Meta-Analysis
Source: J Clin Med. 2024 Apr 7;13(7):2136. doi: 10.3390/jcm13072136 (PMC11012373; doi:10.3390/jcm13072136)
Supplement: Supplementary file 1 [file jcm-13-02136-s001.zip › jcm-2890788-supplementary.pdf]

Database	MEDLINE
Platform	OVID
Date	December 6th 2022
Time	11:12 am
Limits	NONE
Number of Results	204

1 exp Cytomegalovirus Infections/ or Cytomegalovirus/ (37992)
2 (cytomegalovirus* or citomegalovirus* or CMV or HCMV or cytomegalic or cytomegal* or cytomegalo herpetovirus).tw,kw,kf. (57150)
3 ((human or congenital or virus) adj2 (cytomegal* or CMV)).tw,kw,kf. (16252)
4 ("hhv 5" or human herpesvirus or "herpesvirus 5" or "herpesvirus type 5" or "txid10358").tw,kw,kf. (7039)
5 or/1-4 (67855)
6 Viral Load/ or DNA, viral/ or exp polymerase chain reaction/ (567169)
7 DNA Copy Number Variations/ or (copy number polymorphism* or copy number variation* or CNV or dna copy number change* or dna copy number polymorphism* or dna copy number variant* or dna copy number variation*).tw,kw,kf. (24298)
8 ((number* or count or quantit* or amount*) adj3 (virus or viral)).tw,kw,kf. (12629)
9 (viral dna or virus dna or deoxyribonucleic acid virus or virus deoxyribonucleic acid or virus desoxyribonucleic acid or polymerase chain reaction* or PCR or qPCR).tw,kw,kf. (798571)
10 ((anchored or inverse* or nested) adj3 pcr).tw,kw,kf. (14118)
11 ((virus or viral) adj3 (load* or burden* or titer* or titre* or particle count*)).tw,kw,kf. (57039)
12 or/6-11 (1128835)
13 Amniocentesis/ or Amniotic Fluid/ (25280)
14 (amnio or amnios or amniocentes* or amniotic fluid* or amnion centes*).tw,kw,kf. (29280)
15 13 or 14 (36894)
16 5 and 12 and 15 (204)

Database	PubMed
Platform	N/A
Date	December 6th 2022
Time	11:31 am
Limits	NONE
Number of Results	11

(("Cytomegalovirus Infections"[mesh] or "cytomegalovirus"[mesh] or "cytomegal*"[tw] or "citomegal*"[tw] or "cmv"[tw] or "hcmv"[tw] or "hhv 5"[tw] or "hhv5"[tw] or "human herpesvirus"[tw] or "herpesvirus 5"[tw] or "herpesvirus type 5"[tw] or "txid 10358"[tw] or (("human"[tw] or "congenital"[tw] or "virus"[tw] or "viral"[tw]) AND ("cytomegal*"[tw] or "citomegal*"[tw] or "CMV"[tw]))) AND ("Viral Load"[mesh] or "DNA, viral"[tw] or "polymerase chain reaction"[mesh] or "DNA Copy Number Variations"[mesh] or "copy number polymorphism*"[tw] or "copy number variation*"[tw] or "CNV"[tw] or "dna copy number change*"[tw] or "dna copy number polymorphism*"[tw] or "dna copy number variant*"[tw] or "dna copy number variation*"[tw] or "viral dna"[tw] or "virus dna"[tw] or "deoxyribonucleic acid virus"[tw] or "virus deoxyribonucleic acid"[tw] or "virus desoxyribonucleic acid"[tw] or "polymerase chain reaction*"[tw] or "PCR"[tw] or "qPCR"[tw] or (("number*"[tw] or "count"[tw] or "quantit*"[tw] or "amount*"[tw]) AND ("virus"[tw] or "viral"[tw])) or (("anchored"[tw] or "inverse*"[tw] or "nested"[tw]) AND "pcr"[tw]) or (("virus"[tw] or "viral"[tw]) AND ("load*"[tw] or "burden*"[tw] or "titer*"[tw] or "titre*"[tw] or "particle count*"[tw])))) AND ("Amniocentesis"[mesh] or "Amniotic Fluid"[mesh] or "amnio"[tw] or "amnios"[tw] or "amniocentes*"[tw] or "amniotic fluid*"[tw] or "amnion centes*"[tw]) AND pubmednotmedline[sb]

Database	Embase
Platform	OVID
Date	December 6th 2022
Time	11:50 am
Limits	NONE
Number of Results	429

1 exp cytomegalovirus infection/ or exp cytomegalovirus/ (77320)
2 (cytomegalovirus* or citomegalovirus* or CMV or HCMV or cytomegalic or cytomegal* or cytomegalo herpetovirus).tw,kw. (84302)
3 ((human or congenital or virus) adj2 (cytomegal* or CMV)).tw,kw. (19346)
4 ("hhv 5" or human herpesvirus or "herpesvirus 5" or "herpesvirus type 5" or "txid10358").tw,kw. (8017)
5 or/1-4 (112001)
6 exp virus load/ or virus DNA/ or exp polymerase chain reaction/ or copy number variation/ (1302972)
7 (copy number polymorphism* or copy number variation* or CNV or dna copy number change* or dna copy number polymorphism* or dna copy number variant* or dna copy number variation*).tw,kw. (28625)
8 ((number* or count or quantit* or amount*) adj3 (virus or viral)).tw,kw. (16849)
9 (viral dna or virus dna or deoxyribonucleic acid virus or virus deoxyribonucleic acid or virus desoxyribonucleic acid or polymerase chain reaction* or PCR or qPCR).tw,kw. (1099565)
10 ((anchored or inverse* or nested) adj3 pcr).tw,kw. (17498)
11 ((virus or viral) adj3 (load* or burden* or titer* or titre* or particle count*)).tw,kw. (82080)
12 or/6-11 (1662378)
13 exp amniocentesis/ (18075)

14 exp amnion fluid/ (30015)
15 (amnio or amnios or amniocentes* or amniotic fluid* or amnion centes*).tw,kw. (40080)
16 or/13-15 (54409)
17 5 and 12 and 16 (429)

Database	Global Index Medicus
Platform	N/A
Date	December 6th 2022
Time	11:59 am
Limits	NONE
Number of Results	24

(tw:(cytomegalovirus or CMV or HCMV)) AND (tw:(viral load or polymerase chain reaction or pcr or viral dna)) AND (tw:(amniocentes* or amniotic fluid*))

Database	Web of Science
Platform	Clarivate
Date	December 6th 2022
Time	12:28 pm
Limits	NONE
Number of Results	233

((TS=("cytomegalovirus" or "cytomegal*" or "citomegal*" or "cmv" or "hcmv" or "hhv 5" or "hhv8" or "human herpesvirus" or "herpesvirus 5" or "herpesvirus type 5" or "txid 10358" or (("human" or "congenital" or "virus") NEAR/2 ("cytomegal*" or "CMV")))) AND TS=("copy number polymorphism*" or "copy number variation*" or "CNV" or "dna copy number change*" or "dna copy number polymorphism*" or "dna copy number variant*" or "dna copy number variation*" or "viral dna" or "virus dna" or "deoxyribonucleic acid virus" or "virus deoxyribonucleic acid" or "virus desoxyribonucleic acid" or "polymerase chain reaction*" or "PCR" or "qPCR" or (("number*" or "count" or "quantit*" or "amount*") NEAR/3 ("virus" or "viral")) or (("anchored" or "inverse*" or "nested") NEAR/3 "pcr") or (("virus" or "viral") NEAR/3 ("load*" or "burden*" or "titer*" or "titre*" or "particle count*")))) AND TS=("Amniocentes*" or "Amniotic Fluid*" or "amnio" or "amnion" or "amnion centes*")

Database	Cochrane Database of Systematic Reviews + Cochrane Central Database of Controlled Trials
Platform	OVID
Date	December 6th 2022
Time	12:39 pm
Limits	NONE
Search	Run concurrently
Number of Results	13

1 exp Cytomegalovirus Infections/ or Cytomegalovirus/ (868)
2 (cytomegalovirus* or citomegalovirus* or CMV or HCMV or cytomegalic or cytomegal* or cytomegalo herpetovirus).tw,hw. (3276)
3 ((human or congenital or virus) adj2 (cytomegal* or CMV)).tw,hw. (347)
4 ("hhv 5" or human herpesvirus or "herpesvirus 5" or "herpesvirus type 5" or "txid10358").tw,hw. (99)
5 or/1-4 (3338)
6 Viral Load/ or DNA, viral/ or exp polymerase chain reaction/ (5308)
7 DNA Copy Number Variations/ or (copy number polymorphism* or copy number variation* or CNV or dna copy number change* or dna copy number polymorphism* or dna copy number variant* or dna copy number variation*).tw,hw. (1228)
8 ((number* or count or quantit* or amount*) adj3 (virus or viral)).tw,hw. (775)
9 (viral dna or virus dna or deoxyribonucleic acid virus or virus deoxyribonucleic acid or virus desoxyribonucleic acid or polymerase chain reaction* or PCR or qPCR).tw,hw. (19816)
10 ((anchored or inverse* or nested) adj3 pcr).tw,hw. (148)
11 ((virus or viral) adj3 (load* or burden* or titer* or titre* or particle count*)).tw,hw. (8557)
12 or/6-11 (28853)
13 Amniocentesis/ or Amniotic Fluid/ (315)
14 (amnio or amnios or amniocentes* or amniotic fluid* or amnion centes*).tw,hw. (1347)
15 13 or 14 (1347)
16 5 and 12 and 15 (13)

Database	CINAHL
Platform	Ebsco
Date	December 6th 2022
Time	1:27 pm
Limits	NONE
Number of Results	52

S4 S1 AND S2 AND S3
52
S3 (MH "Amniocentesis" or MH "Amniotic Fluid") OR TI ((amnio or amnios or amniocentes* or amniotic fluid* or amnion centes*)) OR AB ((amnio or amnios or amniocentes* or amniotic fluid* or amnion centes*))
5,851
S2 (MH "viral load" or MH "polymerase chain reaction+") OR TI (copy number polymorphism* or copy number variation* or CNV or dna copy number change* or dna copy number polymorphism* or dna copy number variant* or dna copy number variation* or viral dna or virus dna or deoxyribonucleic acid virus or virus deoxyribonucleic acid or virus desoxyribonucleic acid or polymerase chain reaction* or PCR or qPCR or ((number* or count or quantit* or amount*) N3 (virus or viral)) or ((anchored or inverse* or nested) N3 pcr) or ((virus or viral) N3 (load* or burden* or titer* or titre* or particle count*))) OR AB (copy number polymorphism* or copy number variation* or CNV or dna copy number change* or dna copy number polymorphism* or dna copy number variant* or dna copy number variation* or viral dna or virus dna or deoxyribonucleic acid virus or virus deoxyribonucleic acid or virus desoxyribonucleic acid or polymerase chain reaction* or PCR or qPCR or ((number* or count or quantit* or amount*) N3 (virus or viral)) or ((anchored or inverse* or nested) N3 pcr) or ((virus or viral) N3 (load* or burden* or titer* or titre* or particle count*)))
102,236
S1 (MH "Cytomegalovirus Infections+" or MH "Cytomegaloviruses") OR TI (cytomegalovirus* or citomegalovirus* or CMV or HCMV or cytomegalic or cytomegal* or cytomegalo herpetovirus or "hhv 5" or human herpesvirus or "herpesvirus 5" or "herpesvirus type 5" or "txid10358" or ((human or congenital or virus) n2 (cytomegal* or CMV))) OR AB (cytomegalovirus* or citomegalovirus* or CMV or HCMV or cytomegalic or cytomegal* or cytomegalo herpetovirus or "hhv 5" or human herpesvirus or "herpesvirus 5" or "herpesvirus type 5" or "txid10358" or ((human or congenital or virus) n2 (cytomegal* or CMV)))
7,096

Database	Google Scholar
Platform	N/A
Date	December 6th 2022
Time	3:27 pm
Limits	NONE
Number of Results	First 200 results

(cytomegalovirus|CMV|HCMV|"hhv 5"|"herpesvirus 5")(viral load|viral dna|polymerase chain reaction|pcr|dna copy number variation*)(amniocentes*|amniotic fluid*|amnio)

Database	ClinicalTrials.gov
Platform	N/A

Date	December 6th 2022
Time	1:18 pm
Limits	NONE
Number of Results	79

Cytomegalovirus AND amniocentesis (6)
Cytomegalovirus AND amniotic fluid (10)
Cytomegalovirus AND prenatal (27)
Cytomegalovirus AND fetal (29)
Cytomegalovirus AND utero (7)
(79)
